# Positioning accuracy during VMAT of gynecologic malignancies and the resulting dosimetric impact by a 6-degree-of-freedom couch in combination with daily kilovoltage cone beam computed tomography

**DOI:** 10.1186/s13014-015-0412-x

**Published:** 2015-04-26

**Authors:** Lihong Yao, Lihong Zhu, Junjie Wang, Lu Liu, Shun Zhou, ShuKun Jiang, Qianqian Cao, Ang Qu, Suqing Tian

**Affiliations:** Department of Radiation Oncology, Peking University Third Hospital, Hua-yuan North Road No.49, Haidian District, Beijing, 100191 P. R. China

## Abstract

**Background:**

To improve the delivery of radiotherapy in gynecologic malignancies and to minimize the irradiation of unaffected tissues by using daily kilovoltage cone beam computed tomography (kV-CBCT) to reduce setup errors.

**Methods:**

Thirteen patients with gynecologic cancers were treated with postoperative volumetric-modulated arc therapy (VMAT). All patients had a planning CT scan and daily CBCT during treatment. Automatic bone anatomy matching was used to determine initial inter-fraction positioning error. Positional correction on a six-degrees-of-freedom (6DoF) couch was followed by a second scan to calculate the residual inter-fraction error, and a post-treatment scan assessed intra-fraction motion. The margins of the planning target volume (M_PTV_) were calculated from these setup variations and the effect of margin size on normal tissue sparing was evaluated.

**Results:**

In total, 573 CBCT scans were acquired. Mean absolute pre-/post-correction errors were obtained in all six planes. With 6DoF couch correction, the M_PTV_ accounting for intra-fraction errors was reduced by 3.8–5.6 mm. This permitted a reduction in the maximum dose to the small intestine, bladder and femoral head (*P* = 0.001, 0.035 and 0.032, respectively), the average dose to the rectum, small intestine, bladder and pelvic marrow (*P* = 0.003, 0.000, 0.001 and 0.000, respectively) and markedly reduced irradiated normal tissue volumes.

**Conclusions:**

A 6DoF couch in combination with daily kV-CBCT can considerably improve positioning accuracy during VMAT treatment in gynecologic malignancies, reducing the M_PTV_. The reduced margin size permits improved normal tissue sparing and a smaller total irradiated volume.

## Background

Radiation therapy (RT) plays a major role in the management of gynecologic malignancies, especially in the postoperative phase for patients who have one or more pathologic risk factors (e.g. positive lymph nodes, parametrial infiltration, involved surgical margins, large tumor size, deep stromal invasion, or invasion of the lymphovascular space). In these cases, RT is regarded as adjuvant therapy following radical hysterectomy, and has been shown to reduce the risk of recurrence and prolong progression-free survival [1].

Intensity-modulated RT (IMRT) creates steep dose gradients between the tumor target and uninvolved organs at risk (OAR). However, inter- and intra-fractional organ motion, as well as setup uncertainties can result in compromised local tumor control rate and an increased risk of side effects. To ensure enough target volume coverage during RT, the clinical target volume (CTV) is expanded by a safety margin to generate a planning target volume (PTV) [2]. The International Commission on Radiation Units and Measurements (ICRU) defines the internal margin and set-up margin to compensate for these geometric variations and uncertainties. Determining the optimal margin that provides best possible normal tissue sparing in the context of dose escalation to the tumor remains a focus of research.

Improved accuracy in the patient setup procedure can potentially permit the reduction of safety margins [3]. Accurate localization of the tumor position at the time of radiotherapy delivery is therefore of particular importance. Image-guided radiotherapy (IGRT) is a modern method that achieves highly accurate patient setup verification [4-6]. Of the image-guided technologies, kilovoltage cone beam computed tomography (kV-CBCT) is one of the most commonly used methods to provide the volumetric anatomic information, and to evaluate and correct patient positioning before each treatment fraction [7]. A further improvement of the positioning accuracy can be achieved by combining kV-CBCT with a 6-degree-of-freedom (6DoF) couch [6,8-10]. However, to our knowledge, only a few studies have analyzed setup errors for postoperative gynecological cancer patients in the context of a combined IGRT and 6DoF couch.

In our study, all the patients were treated with volumetric-modulated arc therapy (VMAT), which is a form of IMRT that can provide even better sparing of OAR, with fewer monitor units and a lower delivery time than fixed-angle IMRT [11]. Treatment time reduction may play a role in reducing both patients’ discomfort and the potential for intra-fractional motion. The aim of this work is to determine setup errors in six dimensions in the management of postoperative gynecological cancer using daily kV-CBCT, to determine appropriate PTV margins (M_PTV_) in terms of the results obtained, and to evaluate the dose of radiation to OAR and irradiated volume changes on the basis of the appropriate M_PTV_.

## Methods

### Patient characteristics

From January to May 2014, 13 postoperative gynecological cancer patients treated by combined chemotherapy and VMAT at the Peking University Third Hospital were prospectively studied. Of the 13 patients, six were diagnosed with cervical cancer and the remainder had endometrial cancer. The patients were staged according to the International Federation of Gynecology and Obstetrics (FIGO) Surgical Staging Systems for cervical and endometrial carcinoma. All patients were confirmed to be at high risk for relapse after radical hysterectomy with pelvic lymphadenectomy. This study was approved by the Peking University Third Hospital review board and written informed consent was obtained from these patients. The characteristics of the patient cohort are shown in Table 1.

**Table 1:** **Patient characteristics (n = 13)**

**Characteristics**	**No. of patients (%)**
**Age** (years)	
Median	56
Range	39-63
**Histology**	
Squamous cell carcinoma	6 (46.2%)
Adenocarcinoma	7 (53.8%)
**FIGO stage**	
IA	2 (15.4%)
IB	1 (7.7%)
IB1	4 (30.8%)
IIA	3 (23.1%)
IIA1	3 (23.1%)
**Histopathological risk factors**	
Deep stromal invasion ≥1/2	4 (30.8%)
Lymphovascular involvement	7 (53.9%)
Pelvic lymph node metastasis	2 (15.4%)
Parametrial involvement	2 (15.4%)
High grade	10 (77%)
**No. of histopathological risk factors**	
1	4 (30.8%)
2	6 (46.2%)
3	3 (23.1%)

### CT simulation and treatment planning

All patients were instructed to empty the rectum, fill the bladder, and drink 1000 ml water 1 hour before the CT simulation and each treatment fraction. All patients had a CT scan for treatment planning. Patients were immobilized in the supine position using thermoplastic body mask fixation and feetfix (CIVCO, Orange City, IA, USA), with their arms raised above the head and each hand clasping the opposite elbow. The scans ranged from the upper edge of the first lumbar vertebra to 5 cm inferior to the ischial tuberosity, with 5 mm slice thickness and spacing. The images were transferred to the treatment planning system (Oncentra® External Beam v4.3) for contouring and treatment planning. The target volumes were delineated in accordance with the ICRU reports 50 and 62 [2,12] and the consensus guidelines achieved by the Radiation Therapy Oncology Group (RTOG) study 4018 [13]. The PTVs were obtained by using three-dimensional (3D) automatic expansions of CTVs, adding 5 mm in the medio-lateral (ML) and posterior (P) directions, and 8 mm in the superio-inferior (SI) and anterior (A) directions. The prescribed dose was 50.4 Gy to 95% of the PTV in a total of 28 fractions. All patients were treated with one fraction for 5 days per week.

### CBCT acquisition and registration

The kV-CBCT images were acquired using the Elekta Medical Systems linear accelerator equipped with kV imaging capabilities (Axesse™; Elekta Medical Systems). The acquisition parameters were as follows: kVp, 120 kV; nominal milliamperes per frame, 40 mA; kV filter, f1; and gantry rotation, −180°–180°. Before each fraction, patients were positioned first on a 6DoF couch (HexaPOD™ Evo, Elekta-medical intelligence) by aligning room lasers with skin markings, after conventional positioning, and a CBCT scan was acquired on a daily basis. As all the patients in our study had undergone radical surgery, radiation oncologists and therapists evaluated the image online immediately by registering the CBCT scan to the planning CT scan by automatic bone anatomy matching (including the symphysis pubis, sacroiliac joint and the upper edge of the bilateral iliac bone). If the registration was unsatisfactory, the therapists would carry out a further manual registration. The initial inter-fraction setup variations in 3D translational and rotational directions were obtained using Elekta Medical system XVI software. Once the above steps were completed, the 6DoF couch was automatically shifted to correct for any deviation in the ML, SI and AP directions, as well as pitch (rotation around the lateral axis), roll (rotation around the SI axis) and yaw (rotation around the AP axis). In order to measure the residual inter-fraction setup variations and verify the accuracy of the automatic correction, a second CBCT scan was performed. After VMAT delivery, a final CBCT was acquired to assess intra-fractional motion. Both the second and the third CBCT scans were performed twice a week. This CBCT scan arrangement was interrupted occasionally as an unavoidable consequence of heavy treatment workload.

### Setup variation analysis

The deviations between the CBCT and the planning CT in 3D translational and rotational directions were defined as the setup variation. In accordance with the definitions by van Herk [14], variations in RT were classified as random and systematic. For each patient, the mean (M) was used to describe the systematic error, and the standard deviation (SD) represented the random error for the daily fraction measurements. The group systematic error (∑) was defined as the SD of all patients’ M. The group random error (σ) was defined as the root mean square of all patients’ SD.

### PTV margins

PTV margins (M_PTV_) in ML, SI and AP directions were calculated by the geometric margin equation M_PTV_ = 2.5 ∑_total_ + 0.7σ_total_, which ensures that the minimum CTV dose is 95% for 90% patients [15]. The ∑_total_ and σ_total_ were calculated as follows:

$$ {{\displaystyle \sum {{}_{\mathrm{total}}}^2=\left({\displaystyle {\sum}_{\mathrm{inter}\hbox{-} \mathrm{fraction}}}\right)}}^2+{\left({\displaystyle {\sum}_{\mathrm{intra}\hbox{-} \mathrm{fraction}}}\right)}^2,\ {\upsigma_{\mathrm{total}}}^2 = {\left({\upsigma}_{\mathrm{inter}\hbox{-} \mathrm{fraction}}\right)}^2 + {\left({\upsigma}_{\mathrm{intra}\hbox{-} \mathrm{fraction}}\right)}^2 $$

### Dosimetric analysis

The clinical VMAT treatment plan of each patient was termed plan A. The new plan with the addition of M_PTV_ was termed plan B. All beam parameters were kept same for plan A and plan B. In accordance with the intra-fractional setup variation of each patient, we moved only the isocenters of plan B to generate plan C. For each patient, dose volume histograms (DVHs) for plan A and plan B were compared to determine the maximum and average radiation dose to OAR (rectum, small intestine, bladder, femoral head and pelvic bone marrow), as well as the percentage of irradiated volume receiving more than 5, 10, 20, 30 and 40 Gy (V_5_, V_10_, V_20_, V_30_ and V_40_). The plan C was used to evaluate the D_95_ and V_100_ of CTV.

### Statistical analysis

All statistical analysis and processing was conducted using SPSS software, version 19.0 (SPSS, Chicago, IL, USA). Where data were consistent with the normal distribution, statistical analysis was performed using the paired *t*-test; otherwise the Wilcoxon rank sum test was used. A two-tailed *P* value <0.05 was considered statistically significant.

## Results

### Number of CBCT scans

Datasets for a total of 573 CBCT scans were obtained from 13 patients, of which 308 (53.8%) were obtained before the 6DoF couch correction, 137 (23.9%) followed the 6DoF couch correction, and 128 (22.3%) were obtained following VMAT delivery. The median number of scans per patient was 45, and there were no missing scans.

### Evaluation of setup variation

**Figure 1:**
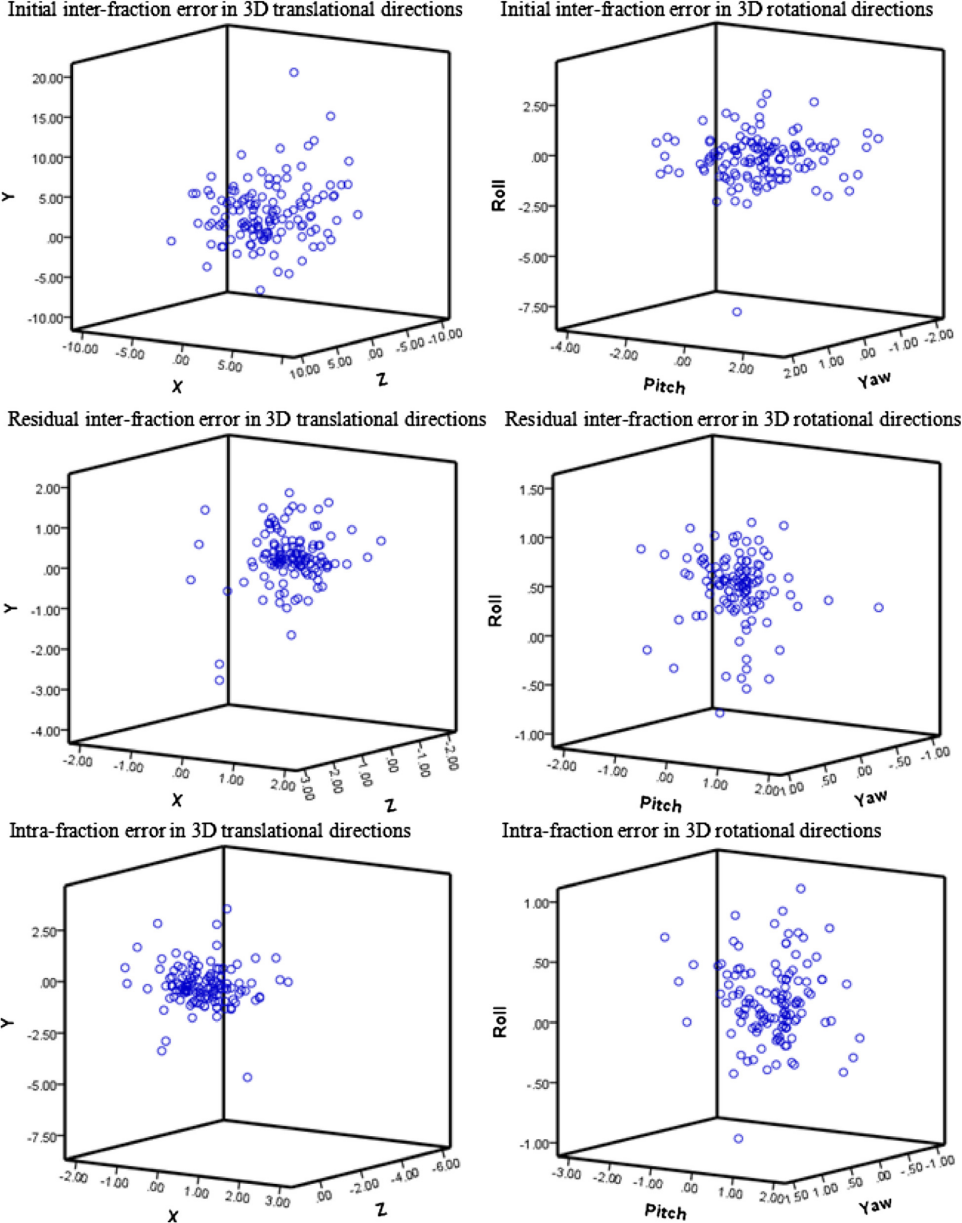
The setup errors throughout the entire course of VMAT for all the patients. The X, Y, and Z axes represent the medial-lateral, superior-inferior and anterior-posterior directions, respectively.

Only those data points with complete initial, residual and post-treatment scans were used to evaluate the inter- and intra-fraction error (Figure 1). Table 2 summarizes the overall setup variation in each direction. The overall translational and rotational inter-fraction displacements were significantly reduced after the corrections of the 6DoF couch. The mean absolute values of the pre-/post-correction errors were 2.52/0.45 mm ML, 3.06/0.45 mm SI, 2.36/0.30 mm AP, 0.95/0.29° pitch, 0.91/0.51° roll, and 0.49/0.15° yaw. The percentage of displacements exceeding ± 2 mm or ± 2° were reduced from 51.6% to 0% ML, 57.0% to 2.3% SI, 49.2% to 0.8% AP, 14.1% to 0.0% pitch, 10.2% to 0.0% roll, and 0.0% to 0.0% yaw.

**Table 2:** **Summary of setup variation in translational and rotational direction (mm) for 13 patients**

	**Initial inter-fraction error**				**Residual inter-fraction error**				**Intra-fraction error**			
**Direction**	**M**	**SD**	**Range**	**PDE**	**M**	**SD**	**Range**	**PDE**	**M**	**SD**	**Range**	**PDE**
ML (mm)	−0.3	3.1	−9.0-7.0	51.6%	0.2	0.6	−1.8-1.8	0%	0.2	0.7	−1.8-2.5	1.6%
SI (mm)	2.0	3.7	−7.8-17.4	57.0%	0.0	0.7	−3.3-1.7	2.3%	0.0	1.1	−6.3-4.0	5.5%
AP (mm)	−1.4	2.7	−9.2-5.5	49.2%	−0.1	0.5	−1.6-3.0	0.8%	−0.4	0.7	−6.0-0.9	2.3%
Pitch (°)	−0.2	1.3	−3.8-2.9	14.1%	−0.1	0.5	−1.8-1.6	0.0%	−0.2	0.6	−2.4-1.5	2.3%
Roll (°)	−0.4	1.2	−8.0-2.5	10.2%	0.4	0.4	−0.8-1.1	0.0%	0.1	0.3	−1.0-1.0	0.0%
Yaw (°)	−0.1	0.6	−1.7-1.6	0.0%	0.1	0.2	−0.7-0.9	0.0%	0.1	0.3	−0.8-1.3	0.0%

*Abbreviations*: *M* overall population mean, *SD* standard deviation, *PDE* percentage of displacements exceeding ± 2 mm or ± 2°.

During treatment delivery, the intra-fraction error increased to a certain extent. The average absolute intra-fraction error values were 0.57 mm ML, 0.72 mm SI, 0.46 mm AP, 0.48° pitch, 0.25° roll, and 0.22° yaw. The ∑ and σ values for the residual inter-fraction were significantly smaller than the initial inter-fraction.

### Evaluation of PTV margins

With the 6DoF couch online correction, the total M_PTV_ accounting for intra-fraction errors was reduced by 3.8–5.6 mm (Table 3). If the initial inter-fractional and intra-fractional setup variation were considered, the total M_PTV_ in ML, SI and AP directions were 7.6, 8.3 and 5.6 mm, respectively, while after the 6DoF couch online correction, the corresponding margins in each of the three directions were only 2.0, 2.9 and 1.8 mm.

**Table 3:** **Calculation of total M**_**PTV**_ **(mm) before and after the correction of the 6DoF couch**

**Directions**	**Pre-correction**			**Post-correction**			**TDBA**
	**∑**_**total**_	**σ**_**total**_	**M**_**PTV**_	**∑**_**total**_	**σ**_**total**_	**M**_**PTV**_	
ML	2.4	2.3	7.6	0.6	0.8	2.0	5.6
SI	2.5	3.1	8.3	0.8	1.1	2.9	5.4
AP	1.6	2.5	5.6	0.5	0.7	1.8	3.8

*Abbreviations*: *∑* the SD of all patients’ M, *σ* the root mean square of all patients’ SD, *TDBA* the differences in M_PTV_ before and after correction, Before the correction, ∑_total_^2^ = (∑_initial inter-fraction_)^2^ + (∑_intra-fraction_)^2^, σ_tota_^2^ = (σ_initial inter-fraction_)^2^ + (σ_intra-fraction_)^2^; After the correction, ∑_total_^2^ = (∑_residual inter-fraction_)^2^ + (∑_intra-fraction_)^2^, σ_total_^2^ = (σ_residual inter-fraction_)^2^ + (σ_intra-fraction_)^2^. Other abbreviations as in Table 2.

### Effect of margin size on normal tissue sparing

**Figure 2:**
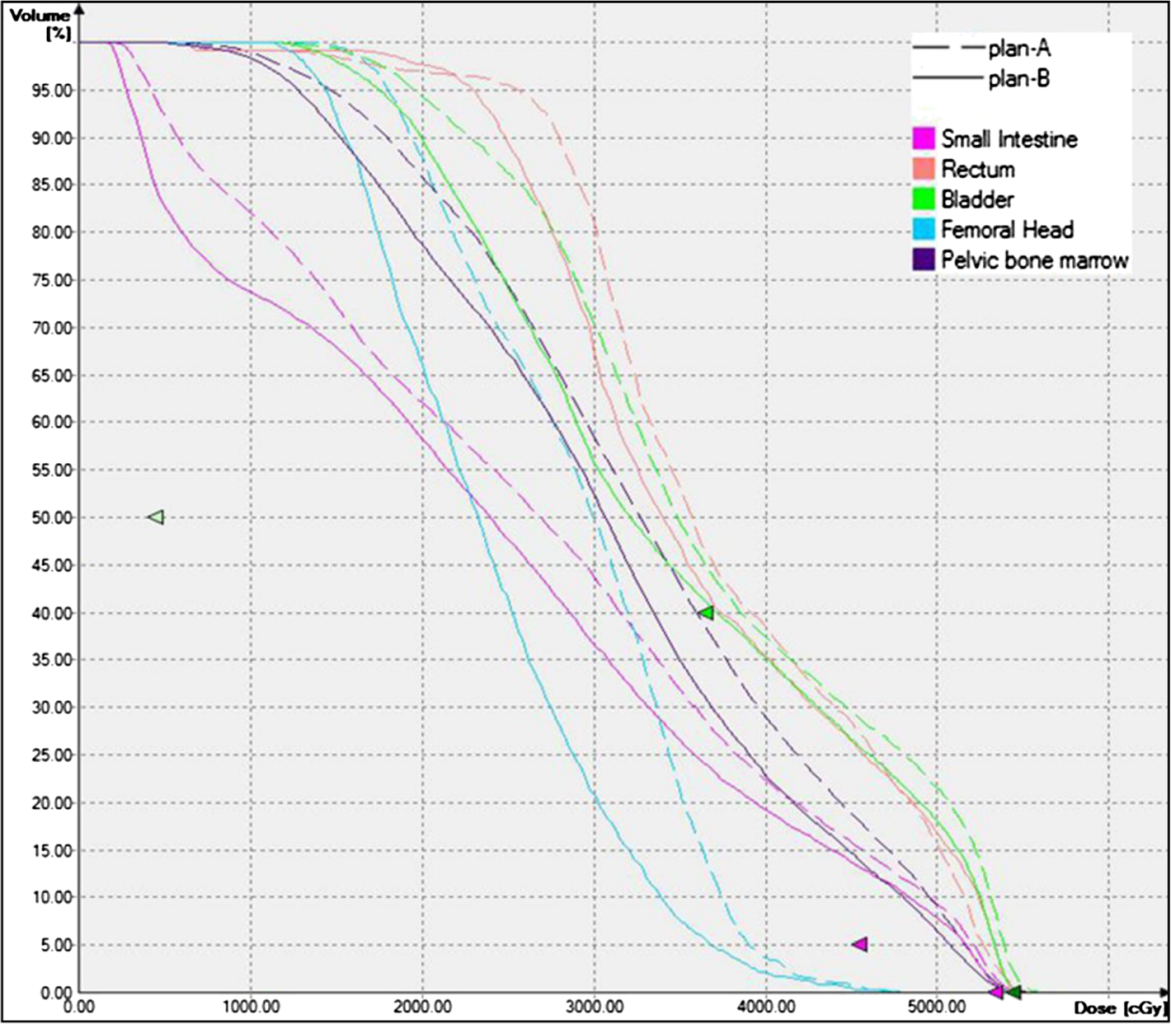
Representative dose-volume histograms for plan A *vs.* plan B.

Using plan C, with the M_PTV_ 2.0, 2.9 and 1.8 mm in ML, SI and AP directions, in the presence of the intra-fraction shifts, D_95_ and V_100_ for the target were 51.5 ± 0.5 Gy and 99.6 ± 0.7% respectively. This ensured the achievement of appropriate dosimetric requirements, such that all the patients had a D_95_ of the prescribed dose. Compared with plan A, plan B significantly reduced the maximum dose for the small intestine, bladder and femoral head (*P* = 0.001, 0.035 and 0.032, respectively), as well as the average dose for the rectum, small intestine, bladder and pelvic bone marrow (*P* = 0.003, 0.000, 0.001 and 0.000, respectively), as summarized in Table 4. Figure 2 displays the dose differences between plan A and plan B. For the rectum, the V_5_, V_10_ and V_40_ calculated using DVHs generated by plan B were lower than those of plan A (*P* = 0.01, 0.014 and 0.000, respectively); for the bladder, V_20_ , V_30_ and V_40_ values were lower with plan B (*P* = 0.036, 0.043 and 0.000, respectively); and for small intestine, V_5_ ,V_10_, V_20_, V_30_ and V_40_ were all significantly reduced (*P* = 0.000, 0.000, 0.000, 0.001, 0.000 and 0.005, respectively). For the pelvic bone marrow, only the V_30_ and V_40_ were lower (*P* = 0.000 and 0.000), while for the femoral head, none of the volume parameters was statistically significantly reduced.

**Table 4:** **Comparison of the OAR radiation dose by the two plans (M ± SD)**

**OAR**	**Plan**	**The maximum dose (Gy)**	**The average dose (Gy)**	**V**_**5**_ **(%)**	**V**_**10**_ **(%)**	**V**_**20**_ **(%)**	**V**_**30**_ **(%)**	**V**_**40**_ **(%)**
Rectum	A	54.4 ± 0.7	35.1 ± 2.5	96.6 ± 4.1	93.4 ± 6.3	89.7 ± 3.2	66.1 ± 11.5	36.6 ± 6.5
	B	54.2 ± 0.6	34.3 ± 2.5	95.0 ± 4.9	91.5 ± 6.3	88.0 ± 8.5	63.8 ± 9.4	34.6 ± 7.2
Small intestine	A	54.5 ± 1.2	28.3 ± 4.9	95.2 ± 5.5	87.1 ± 9.3	70.4 ± 14.7	44.1 ± 16.8	22.3 ± 9.9
	B	53.6 ± 0.8	26.2 ± 4.6	91.6 ± 7.8	83.0 ± 9.4	65.8 ± 14.5	39.1 ± 15.9	18.2 ± 7.5
Bladder	A	54.8 ± 1.1	35.4 ± 1.4	100.0 ± 0.0	100.0 ± 0.1	94.9 ± 4.5	60.5 ± 10.4	34.5 ± 2.5
	B	54.3 ± 0.7	34.4 ± 1.2	100.0 ± 0.0	99.9 ± 0.5	92.1 ± 5.1	57.2 ± 7.5	32.3 ± 2.7
Femoral head	A	47.1 ± 3.1	27.1 ± 3.3	99.8 ± 0.6	98.6 ± 3.1	80.5 ± 16.8	37.7 ± 15.1	4.2 ± 3.5
	B	46.1 ± 3.6	26.4 ± 3.0	99.8 ± 0.8	98.4 ± 4.6	79.2 ± 15.6	31.9 ± 13.2	3.1 ± 2.3
Pelvic BM	A	55.2 ± 1.6	33.3 ± 2.2	100.0 ± 0.0	98.9 ± 1.0	81.3 ± 17.0	58.2 ± 8.0	28.6 ± 6.5
	B	54.6 ± 0.9	31.5 ± 2.1	99.9 ± 0.2	98.7 ± 1.1	81.4 ± 5.6	52.3 ± 8.8	23.4 ± 4.8

*Abbreviations*: *Pelvic BM* pelvic bone marrow, *OAR* uninvolved organs at risk. Other abbreviations as in Tables 2 and 3.

## Discussion

IGRT enhances the precision of patient positioning, allowing for improved sparing of normal tissues through a reduction in treatment margins. Using tighter margins, however, requires the techniques to guarantee precise target localization. In our study, we evaluated the variations in patient setup in the postoperative treatment of gynecological cancers throughout the entire course of VMAT treatment. Compared to previous studies, our study utilized daily kV-CBCT in combination with a 6DoF couch to correct setup errors in 3D translational and rotational directions before delivering each treatment fraction. To eliminate inter-observer variations, automatic registration strategies using bone anatomy recognition were employed. This is one of few studies that analyze the initial positioning, residual inter-fraction error and intra-fraction error for postoperative patients with gynecologic malignancies during VMAT. In addition, we have also evaluated the radiation dose to OAR and the irradiated volume changes on the basis of the appropriate M_PTV_.

In most similar studies, planning CT slice thickness was 3 mm [10,16,17] and 3.75 mm [17], however, we prefer to have this slice thickness for 5 mm in our conventional treatment. This seems to have slightly effect on the evaluation.

The inter-fraction error observed in our study is generally consistent with other reports examining setup errors in pelvic RT of gynecological cancer patients [16-21]. Stromberger et al. [17] analyzed setup errors in gynecologic malignancy treatment by matching the daily megavoltage CT (MVCT) with the planning CT. This group found that the total systematic deviations had means of 0.5, 0.5, −2.0 mm and −0.5° in ML, SI, AP and roll directions, respectively, while the total random deviations were 6.1, 3.4, 3.8 mm and 0.9°, respectively. Santanam et al. [16] also investigated setup uncertainties in two groups of gynecological cancer patients, with one group of 10 patients in whom two-dimensional planar KV and portal MV imaging was used, being contrasted with another group of 10 patients who underwent MVCT imaging. In the planar imaging group, the SD of systematic errors were 0.37, 0.36 and 0.24 cm in ML, SI and AP directions, respectively, while the SD of random error was 0.33, 0.38 and 0.31 cm, respectively. The average shifts per patient were −0.27–1.4 cm ML, −1.2–1.5 cm SI, −1.2–1.0 cm AP, respectively. In the MVCT group, the SD of systematic error was 0.20, 0.46 and 0.15 cm in ML, SI and AP directions, respectively; the SD of the random error was 0.34, 0.48 and 0.37 mm, respectively; and the average shifts per patient were −1.3–1.0 cm ML, −4.1–2.2 cm SI, −0.9–1.2 cm AP. Using daily CBCT scan on-line registration, Laursen et al. [18] reported the SD of systematic error was 2.9, 2.6 and 3.6 mm in ML, SI and AP directions, respectively, and the SD of random error was 3.2, 2.4 and 3.6 mm, respectively. Using off-line registration, the mean and standard deviation for the residual rotational errors were 0.04 ± 1.4° pitch, 0.04 ± 0.9° roll, and −0.06 ± 0.9° yaw. Ahmad et al. [10] reported setup errors in 15 prone-treated cervical cancer patients using the 6D positioning device, 2D EPID and CBCT (acquired twice a week). Patients’ residual overall mean setup variations measured by CBCT imaging were −0.7 mm ML, −1.1 mm SI, 0.1 mm AP, 1.4° pitch, −0.5° roll, and 0.4° yaw, and by EPID imaging were 0.2 mm ML, −0.2 mm SI, 0.2 mm AP, −0.3° pitch, and 0.5° yaw.

The intra-fraction variations caused by organ deformation and/or motion is smaller compared with the inter-fraction variations, but is still of relevance in clinical practice. Although patients were given detailed instruction to empty the rectum and fill the bladder to achieve the purpose of greater sparing of the small bowel and protection of the bladder, a series of studies have reported vaginal motion, and bladder and rectal volume changes during RT [21-29], which can lead to movement of the target volume outside the radiation field and increased exposure of the OAR to high doses of radiation. Santanam’s study [16], also measured shifts on five patients (280 images) during treatment in the planar imaging group, reporting setup errors with a mean and standard deviation of 0.008 ± 0.024 mm ML, 0.018 ± 0.026 mm SI, and −0.031 ± 0.033 mm AP. Jürgenliemk-Schulz et al. [22] used MRI before treatment and weekly during EBRT delivery for cervical and endometrial cancer patients after hysterectomy. They found vaginal CTVs changed their position in the pelvis during time with a maximum in AP direction; this was only weakly related to the volume of the rectum and bore no relationship to the volumes of the other parts of the bowel and the bladder. Although relatively small, these shifts may cause either a geographical miss of the target that will inevitably compromise the tumor local control [23], or unnecessary OAR inclusion into high dose regions that will increase the risk of complications [14].

To ensure adequate target volume coverage while increasing OAR sparing, individualized M_PTV_ may be used [24]. Many factors, including different patient immobilization positions (prone or supine), varying measurement methods (EPDI, kVCBCT, MVCT, MRI) or measurement frequencies (daily or weekly), and differing treatment styles (3DCRT, IMRT, VMAT) can all influence decisions regarding the optimal PTV boundary position, and in the treatment of gynecologic malignancies, a wide range of different M_PTV_ may be observed in the published literature [18,21]. One study using daily MVCT images with online correction before treatment and no immobilization device for patients with pelvic malignancies calculated the CTV-to-PTV margins at 8.3 mm [21]. By contrast, Laursen et al. [18] calculated the margins of 9.6 mm ML, 8.2 mm SI, 11.6 mm AP taking only the initial inter-fraction error into consideration. Though margins that account for rotations are of particular importance, there is no established technique to form a margin that accounts for rotational errors. In our study, however, rotational errors by the 6DoF device correction were significantly reduced. If the initial inter-fractional and intra-fractional setup variation were considered, the total M_PTV_ in ML, SI and AP directions were 7.6, 8.3 and 5.6 mm, respectively, while after the 6DoF couch online correction, the corresponding margins in each of the three directions were only 2.0, 2.9 and 1.8 mm.

Unsurprisingly, gastrointestinal (GI) and genitourinary (GU) toxicity and bone marrow suppression are among the most common side effects in patients with gynecologic malignancies undergoing RT. The use of concurrent chemoradiotherapy for the treatment of high risk postoperative gynecological cancer may increase the incidence and severity of adverse reactions and may reduce patients’ quality of life (in turn affecting the likelihood of treatment proceeding without complication or delay). Compared with conventional whole pelvic radiotherapy in gynecology patients, IMRT is associated with fewer acute GI and GU sequelae, since it can reduce the absolute volume of rectal wall, bladder and bowel irradiated at the prescribed dose [30-33]. In theory, appropriate target margins can also improve the therapeutic gains. A study conducted by Ahamad et al. [25] compared IMRT after hysterectomy with standard conformal treatment on the influence of margin size and the reduction in small bowel radiation dose. They demonstrated the importance of relatively small expansions of the target volume, accurate target delineation, and highly reproducible immobilization. Some authors recommend the use of a bellyboard in postoperative pelvic RT for gynecological cancer patients [26], asserting that treating these patients in the prone position using the bellyboard could reduce the volume of irradiated small bowel and minimize daily setup variations. Our findings show that the irradiated normal tissue volumes were markedly reduced especially for the small intestine and bladder during the VMAT for the gynecological cancer patients, by expanding the CTV to PTV boundary according to the results calculated in this study.

## Conclusions

In summary, a 6DoF couch in combination with daily kV-CBCT can considerably improve the accuracy of patient positioning during VMAT treatment for gynecologic malignancies, with a reduction in the M_PTV_. Normal tissue sparing was enhanced by the effects on margin size, with a reduction in radiation dose to critical organs and smaller total irradiated volume. Together, these finding suggest that the strategy we report could improve outcomes for patients undergoing radiotherapy for gynecologic cancer.

## Abbreviations

KV-CBCT: Kilovoltage cone beam computed tomography
VMAT: Volumetric-modulated arc therapy
6DoF: Six-degrees-of-freedom
M_PTV_: Planning target volume
RT: Radiation therapy
IMRT: Intensity-modulated RT
OAR: Organs at risk
CTV: Clinical target volume
PTV: Planning target volume
ICRU: the International Commission on Radiation Units and Measurements
IGRT: Image-guided radiotherapy
FIGO: the International Federation of Gynecology and Obstetrics
3D: Three-dimensional
ML: Medio-lateral
SI: Superio-inferior
AP: Anterio -posterior
M: Overall population mean
SD: Standard deviation
DVHs: Dose volume histograms
PDE: Percentage of displacements exceeding ± 2 mm or ± 2°
TDBA: The differences in M_PTV_ before and after correction
MVCT: Megavoltage CT
GI: Gastrointestinal
GU: Genitourinary
